# Myotubularin-related proteins regulate KRAS function by controlling plasma membrane levels of polyphosphoinositides and phosphatidylserine

**DOI:** 10.1101/2024.01.22.576612

**Published:** 2024-01-23

**Authors:** Karen M. Henkels, Taylor E. Miller, Ali Naji, Ransome van der Hoeven, Hong Liang, Yong Zhou, Gerald R.V. Hammond, John F. Hancock, Kwang-jin Cho

**Affiliations:** 1 Department of Biochemistry and Molecular Biology, Boonshoft School of Medicine, Wright State University, Dayton, Ohio 45435, USA; 2 Department of Diagnostic and Biomedical Sciences, School of Dentistry, The University of Texas Health Science Center at Houston, Houston, Texas 77030, USA; 3 Department of Integrative Biology and Pharmacology, McGovern Medical School, University of Texas Health Science Center, Houston, Texas 77030, USA; 4 Department of Cell Biology, University of Pittsburgh School of Medicine, Pittsburgh, PA 15213, USA

**Keywords:** Myotubularin-related proteins, phosphatidylserine, KRAS, plasma membrane, phosphatidylinositol, phosphatidylinositol 3-phosphate, phosphatidylinositol 3, 5-bisphosphate, phosphatidylinositol 4-phosphate, oxysterol-binding protein related protein (ORP) 5 and 8

## Abstract

KRAS is a small GTPase, ubiquitously expressed in mammalian cells, that functions as a molecular switch to regulate cell proliferation and differentiation. Oncogenic mutations that render KRAS constitutively active occur frequently in human cancers. KRAS must localize to the plasma membrane (PM) for biological activity. KRAS PM binding is mediated by interactions of the KRAS membrane anchor with phosphatidylserine (PtdSer), therefore, depleting PM PtdSer content abrogates KRAS PM binding and oncogenic function. From a genome-wide siRNA screen to search for genes that regulate KRAS PM localization, we identified a set of phosphatidylinositol (PI) 3-phosphatase family members: myotubularin-related (MTMR) proteins 2, 3, 4 and 7. Here we show that knockdown of *MTMR 2/3/4/7* expression disrupts KRAS PM interactions. The molecular mechanism involves depletion of PM PI 4-phosphate (PI4P) levels, which in turn disrupts the subcellular localization and operation of oxysterol-binding protein related protein (ORP) 5, a PtdSer lipid transfer protein that maintains PM PtdSer content. Concomitantly, silencing *MTMR 2/3/4/7* expression elevates PM levels of PI3P and reduces PM and total cellular levels of PtdSer. In summary we propose that the PI 3-phosphatase activity provided by MTMR proteins is required to generate PM PI for the synthesis of PM PI4P, which in turn, promotes the PM localization of PtdSer and KRAS.

## Introduction

H-, N- and KRAS proteins are small GTPases that oscillate between inactive GDP-bound and active GTP-bound conformational states and operate in signaling cascades that control cell growth and proliferation (Gorfe & Cho, 2019; Henkels et al, 2021). Consistent with this key regulatory role, activating mutations of RAS are present in ~20% of human cancers, with the majority occurring in the KRAS isoform (Prior et al, 2020). All RAS isoforms must be localized to the inner leaflet of the plasma membrane (PM) and be spatially organized into nanoclusters for biological activity (Gorfe & Cho, 2019; Henkels et al., 2021). Membrane interactions are mediated by the RAS C-terminal membrane anchor (Zhou et al, 2017). In the case of KRAS4B, the major expressed splice variant of KRAS (Hood et al, 2023) (hereafter KRAS), this anchor comprises a C-terminal farnesyl cysteine methyl ester, generated by posttranslational modification, and an adjacent polybasic domain of six continuous lysines (Gutierrez et al, 1989; Hancock et al, 1991; Hancock et al, 1989; Hancock et al, 1990). Recent work has shown that the KRAS membrane anchor encodes exquisite binding specificity for phosphatidylserine (PtdSer) lipids with one saturated and one desaturated acyl chain (Zhou et al., 2017). This lipid binding specificity is effectively hard wired into the anchor structure and renders KRAS PM binding, nanoclustering and hence biological function absolutely dependent on PM PtdSer content (Cho et al, 2012b; Cho et al, 2013; van der Hoeven et al, 2013; van der Hoeven et al, 2018).

PtdSer is synthesized in the ER and delivered to the PM against a concentration gradient by the lipid transfer proteins (LTPs), oxysterol-binding protein-related protein (ORP) 5 and 8, operating at ER-PM membrane contact sites. One molecule of phosphatidylinositol (PI) 4-phosphate (P), generated by PM localized PI 4-kinase IIIα (PI4KA) from PI, is exchanged by ORP5/8 for one molecule of ER localized PtdSer (Chung et al, 2015; Moser von Filseck et al, 2015). PI4P delivered to the ER is converted to PI by the SAC1 phosphatase, which operating together with PI4KA, maintains the PI4P gradient that in turn, drives PtdSer transport to the PM (Chung et al., 2015; Moser von Filseck et al., 2015). Concordantly genetic and pharmacological studies have shown that inhibition or loss of any component of the PtdSer transport machinery, including PI4KA, EFR3A (the PM anchor protein for the kinase), ORP5, ORP8 and SAC1 results in a reduction in PM PtdSer content, displacement of KRAS from the PM and abrogation of KRAS signaling (Gulyas et al, 2017; Kattan et al, 2019; Kattan et al, 2021; Sohn et al, 2018). Other pharmacological approaches to deplete PM PtdSer also dissociate KRAS from the PM (Cho et al., 2012b; Garrido et al, 2020; Miller et al, 2019; Tan et al, 2019; Tan et al, 2018; van der Hoeven et al., 2018). The PM is a dynamic organelle, thus KRAS on endomembranes is sequestered by a chaperone protein, phosphodiesterase 6δ, and released to perinuclear membranes in the ARL2/3-dependent manner. The negative charge on the recycling endosome membranes then electrostatically traps KRAS, from where vesicular transport returns KRAS to the PM (Chandra et al, 2012; Schmick et al, 2014). KRAS phosphorylation at the Ser181 residue can disrupt this electrostatic interaction, depleting KRAS from the PM (Cho et al, 2016a; Kovar et al, 2020).

In this context we carried out a cell-based high content screen using a human genomic short interfering (siRNA) library to identify additional potential regulators of KRAS PM localization. Among the hits from the assay were multiple genes that encode proteins with phosphatase activity. Four of these genes encode myotubularin-related (MTMR) proteins 2, 3, 4 and 7, which have 3-phosphatase activity towards phosphatidylinositol (PI) 3-phosphate (P) and PI(3,5)-bisphosphate (PI(3,5)P_2_) (Robinson & Dixon, 2006). In this study, we examined the role of MTMR proteins in the PM localization of KRAS. Our data demonstrate that knockdown (KD) of MTMR 2, 3, 4 or 7 dissociates KRAS, but not HRAS from the PM. We show *inter alia* that the molecular mechanism involves depletion of the PI4P and PtdSer content of the PM, extending the repertoire of proteins required to support the operation of the ORP5/8 LTPs and hence sustain KRAS PM localization.

## Method and Material

### Plasmids and reagents.

shRNA glycerol sets for MTMR2 (RHS4533-EG8898), MTMR3 (RHS4533-EG8897), MTMR4 (RHS4533-EG9110), MTMR7 (RHS4533-EG9108), and human cDNA of MTMR2 (MHS6278–202760229), MTMR3 (MHS6278–202800229), MTMR4 (MHS6278–202801286) and MTMR7 (MHS6278–211688199) were purchased from GE Healthcare Dharmacon. Antibodies were purchased for detecting MTMR2 (PA5–22748; 1:1,000) from Invitrogen, MTMR3 (sc-393779; 1:1,000) from SCBT and MTMR7 (AB150458; 1:1,000) from Abcam. Anti-ppERK (4370; 1:3,000), anti-pAkt (S473) (4060; 1:1,000), anti-total ERK (4696; 1:1,000), anti-total Akt (2920; 1:1,000) antibodies for immunoblotting were from Cell Signaling Technology. Goat anti-mouse IgG (G21040; 1:2,000), anti-rabbit IgG secondary antibodies (G21234; 1:5,000) and CellMask Deep Red plasma membrane stain (C10046; 1:5,000) were purchased from Invitrogen. Antibodies for detecting β-actin (60008–1-Ig; 1:5,000) and GFP (66002–1-lg; 1:4,000) were purchased from Proteintech. Staurosporine (BIA-S1086) was purchased from BioAustralis.

### Generating T47D stable cell lines.

T47D cells (ATCC HTB-13) were maintained in RPMI-1640 medium (30–2001, ATCC) supplemented with 10 μg/mL human recombinant insulin (12585–014, Gibco), 2 mM L-glutamine (CA009–010, GenDepot) and 10% FBS (16000044, Life Technologies). Cells were transfected with mGFP-KRASG12V, -HRASG12V or -LactC2 in pEF6 vector (Invitrogen) using *Trans*IT-BrCa Transfection Reagent (MIR 5500, Mirus), and selected with 10 μg/mL blasticidin for 3 weeks. After the selection, cells were maintained in 5 μg/mL blasticidin. For generating MTMR knockdown cell lines, wild-type (WT) T47D cells were infected with lentivirus expressing shRNA targeting *MTMR2, 3, 4* or *7*. Cells were selected by puromycin (1 μg/mL) for 1 week and maintained in 0.5 μg/mL puromycin. Cells were grown at 37°C in 5% CO_2_ and frequently tested for mycoplasma using MycoAlert Mycoplasma Detection Kit (Lonza, LT07–318).

### Preparing T47D cells for imaging.

5 × 10^5^ T47D cells were seeded on a coverslip on day 1 in RPMI-1640 medium containing 10% FBS, 2mM L-glutamine and 10 μg/mL insulin (complete growth medium). On day 2, fresh complete growth medium was supplemented. On day 3, cells were fixed with 4% PFA for 30 min, quenched with 50 mM NH_4_Cl for 10 min and mounted with Vectashield (H-1000, Vector Laboratories). For CellMask staining, cells were incubated with CellMask stain (1:5,000) for 1 h in complete growth medium and fixed with 4% PFA.

### Lipidomic analysis.

T47D cells stably expressing mGFP-KRASG12V with MTMR 2, 3, 4 or 7 knockdown were cultured in complete growth medium containing 0.5 μg/ml puromycin. Triplicate samples of 1×10^6^ cells were prepared in 333 μL Dulbecco’s PBS (DPBS without Ca^2+^ and Mg^2+^, 14190144, Invitrogen). Lipid extraction and analysis using electron spray ionization and MS/ MS were performed at Lipotype GmbH (Dresden, Germany), as described previously (Gerl et al, 2012; Sampaio et al, 2011). Automated processing of acquired mass spectra, identification, and quantification of detected lipid species were done by LipidXplorer software. Only lipid identifications with a signal-to-noise ratio >5, an absolute abundance of at least 1 pmol, and a signal intensity 5-fold higher than in corresponding blank samples were considered for further data analysis. The abundance of lipids is represented as a heat map with log_2_ scale relative to control (scrambled shRNA) cells.

### Western blotting.

Preparation of cell lysates and immunoblotting were performed as described previously (Cho et al, 2011). Briefly, cells were washed twice with ice-cold 1x phosphate-buffered saline (PBS). Cells were harvested in lysis buffer B containing 50 mM Tris-Cl (pH 7.5), 75 mM NaCl, 25 mM NaF, 5 mM MgCl2, 5 mM EGTA, 1 mM DTT, 100 μM NaVO4, 1% NP-40 plus protease and phosphatase inhibitors. SDS-PAGE and immunoblotting were generally performed using 20–25 μg of lysate from each sample group. Signals were detected by enhanced chemiluminescence (Cat# 34578 and 34075, Thermo Fisher Scientific) and imaged using an Amersham Imager 600 (GE Healthcare). ImageJ software (v1.52) was used to quantify band intensity.

### Validating MTMR4 knockdown.

mRNA was extracted from T47D cells infected with lentivirus expressing shRNA targeting MTMR4 using the RNeasy kit (74104, Qiagen) according to the manufacturer’s instructions. 2 μg of total RNA was converted to cDNA using Superscript II reverse transcriptase (18064–014, ThermoFisher). To verify knockdown, forward and reverse primers specific for human MTMR4 exons 1 and 5, and GAPDH (glyceraldehyde-3-phosphate dehydrogenase) exons 1 and 3 were designed: for MTMR4, 5’-CCAAGCCAAGGATCTGTTCCC-3’ and 5’-TGTGTGAGACTCTCCAGACGT-’3’, and for GAPDH, 5’- GGAGCGAGATCCCTCCAAAAT-3’ and 5’-GGCTGTTGTCATACTTCTCATGG-3’, respectively. cDNA was amplified by PCR (P2311, GenDepot) and the products were resolved by 1.5% agarose gel electrophoresis and visualized by ethidium bromide staining.

### *C. elegans* vulva quantification assay.

*let-60* (n1046) worms were kindly provided by Swathi Arur (MD Anderson Cancer Center, Houston, TX). RNAi was induced by feeding *let-60* worms through adult stage with *E. coli* HT115, producing double-stranded RNA to target genes. The presence of the multivulva phenotype was scored using a DIC/Nomarski microscope. All RNAi clones were from the *C. elegans* RNAi (Ahringer) collection (Source BioScience) and were sequenced.

### Lysenin staining.

A maltose binding protein (MBP)-GFP-lysenin fragment (amino acid residues 161 to 297) was purified as previously described (Maekawa et al, 2016). WT T47D cells were fixed with 4% PFA, and permeabilized with 0.05% saponin for 30 min at room temperature (RT), followed by labeling with (15 μg/mL) MBP-GFP-lysenin for 15 min at RT. Cells were quenched with 50 mM NH_4_Cl in the dark for 10 min, and further labeled with DAPI (2.5 μg/mL) in the dark for 10 min.

### Proliferation assay.

T47D cells stably expressing mGFP-KRASG12V and MTMR 2, 3, 4 or 7 knockdown were seeded at 1 ×10^4^ in triplicate onto a 96-well plate in complete growth medium containing 0.5 μg/mL puromycin. Cells were cultured for 4 days. Fresh complete growth medium containing 0.5 μg/mL puromycin was supplemented every 24 h. On day 5, cell proliferation was assayed by counting cells using Countess II cell counter (Invitrogen).

### Annexin V binding assay.

MTMR 2, 3, 4 or 7 knockdown T47D cells stably expressing mGFP-KRASG12V were incubated with Cy5-conjugated annexin V (559934, BD Pharmingen) according to the manufacturer’s instructions. Annexin V-positive cells were counted using a BD AccuriC6 Analyzer with a 670LP nm filter. mGFP-KRASG12V T47D cells expressing scrambled shRNA were treated with 1 μM staurosporine for 6h to induce apoptosis.

### Electron microscopy.

Plasma membrane sheets were prepared from Caco-2 or T47D cells expressing GFP-tagged proteins of interest and fixed as previously described (Hancock & Prior, 2005; Prior et al, 2003a; Prior et al, 2003b). For univariate analysis, plasma membrane sheets were labeled with anti-GFP antibody conjugated to 4.5-nm gold particles. Digital images of the immunogold-labeled plasma membrane sheets were taken in a transmission electron microscope. Intact 1 μm^2^ areas of the plasma membrane sheet were identified using ImageJ software, and the (x, y) coordinates of the gold particles were determined (Hancock & Prior, 2005; Prior et al., 2003a; Prior et al., 2003b). K-functions (Ripley, 1977) were calculated and standardized on the 99% confidence interval (CI) for univariate functions (Diggle, 2000; Hancock & Prior, 2005; Prior et al., 2003a; Prior et al., 2003b). In the case of univariate functions, a value of L(r)—r greater than the CI indicates significant clustering, and the maximum value of the function (Lmax) estimates the extent of clustering. Differences between replicated point patterns were analyzed by constructing bootstrap tests as described previously (Diggle, 2000; Plowman & Hancock, 2005), and the statistical significance against the results obtained with 1,000 bootstrap samples was evaluated.

## Results

### Genome-wide siRNA screening identifies novel genes regulating KRAS PM interaction.

To identify novel regulators of KRAS PM interaction, we performed an image-based high content screen of a human genomic siRNA library. A Caco-2 (human colorectal adenocarcinoma) cell line stably expressing monomeric green fluorescent protein (GFP)-tagged oncogenic mutant KRAS (KRASG12V) was generated and transfected with siRNA pools comprising four different oligonucleotides against each gene. Four days after transfection, cells were imaged using an automated confocal microscope to analyze the extent of KRAS mislocalization from the PM. We then narrowed the list of hits using a counter screen against HRASG12V to identify KRASG12V-specific mediators. Bioinformatics analysis revealed that 8 of these putative KRASG12V-specific mediators encode proteins with phosphatase activity (Table 1), 4 of which are members of the myotubularin-related (MTMR) protein family. There are 14 human myotubularins, which exhibit 3-phosphatase activity towards PI3P and PI(3,5)P_2_, generating PI and PI5P, respectively (Robinson & Dixon, 2006). MTMR proteins have previously been shown to regulate many cellular processes including endocytosis, membrane trafficking, cell proliferation, autophagy, cytokinesis and cytoskeletal dynamics (Hnia et al, 2012). We selected this *MTMR* gene set for further analysis.

### MTMR 2/3/4/7 regulate the PM localization of KRASG12V, but not HRASG12V.

**Figure 1.**
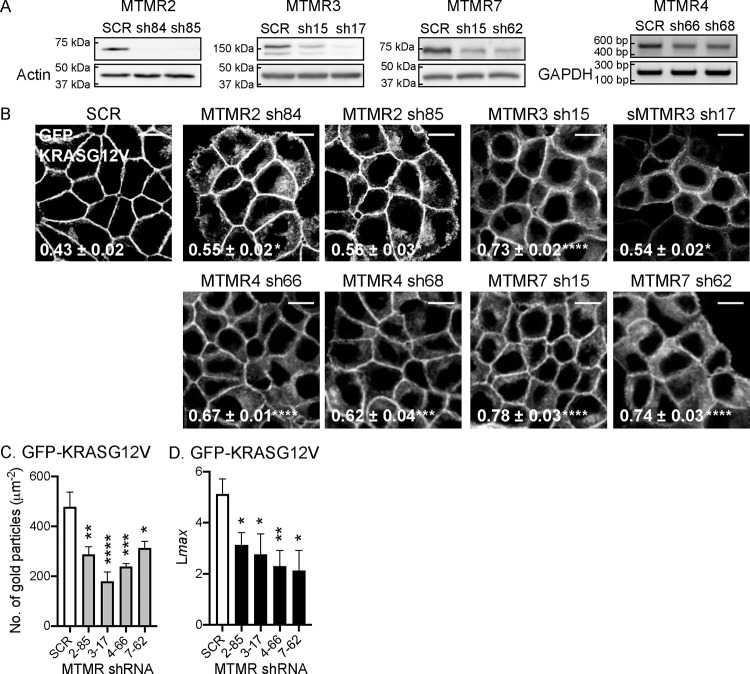
MTMR 2/3/4/7 regulate the PM localization of KRASG12V. (**A**) T47D cells stably expressing GFP-KRASG12V were infected with lentivirus expressing scrambled shRNA (SCR) or shRNA targeting *MTMR2, 3, 4* or *7*, followed by 1 ug/mL puromycin selection. Cell lysates were prepared and immunoblotted with anti-MTMR2, 3 or 7 antibodies. Actin blots are shown as loading controls. For MTMR4, cDNA was amplified with primers specific for MTMR4 exons 1 and 5, or GAPDH exons 2 and 3 as a loading control. (**B**) These cells were incubated with CellMask for 1 h at 37°C incubator, fixed with 4% PFA and imaged by confocal microscopy. Representative GFP-KRASG12V images are shown. Their corresponding CellMask and merged images are shown in Fig. S2. The inserted values represent a mean fraction ± S.E.M. of CellMask colocalizing with GFP-KRASG12V calculated by Manders’ coefficient from three independent experiments. Scale bar – 10 μm. (**C**) Intact basal PM sheets prepared from T47D cells co-expressing GFP-KRASG12V and shRNA targeting *MTMR2, 3, 4* or *7* were labeled with anti-GFP-conjugated gold particles and visualized by EM. Representative EM images are shown in Fig. S3. The graphs show a mean number of gold particles ± SEM (n ≥ 15). (**D**) Spatial mapping of the same gold-labeled PM sheets was performed. The peak values, L*max*, representing the extent of KRAS spatial organization, are shown as bar graphs (n ≥ 15). Significant differences between control (SCR-expressing) and *MTMR*-silenced cells were assessed by one-way ANOVA test for (B and C) and bootstrap tests for (D) (* p < 0.05, ** p < 0.01, *** p < 0.001, **** p < 0.0001).

To validate the siRNA screen, we generated short-hairpin RNA (shRNA)-mediated stable KD cell lines. T47D (human mammary gland ductal carcinoma) epithelial cells stably expressing GFP-KRASG12V were infected with lentiviruses expressing shRNA targeting *MTMR2, 3, 4* and *7*, followed by puromycin selection. For each target gene, we tested 4 different shRNAs and selected the two most effective KD for further experiments. Immunoblotting verified that endogenous expression levels of MTMR2, 3 and 7 were reduced by the cognate shRNAs (Fig. 1A and S1). MTMR4 mRNA levels were also reduced by shRNA expression; we evaluated mRNA because the anti-MTMR4 antibodies we tested were not able to detect endogenous MTMR4 (Fig. 1A). To study KRAS cellular localization cells were incubated with CellMask, a dye staining cellular membranes (Hu et al, 2013; Maechler et al, 2019; Monkemoller et al, 2015; Park et al, 2019), and imaged by confocal microscopy. To quantify the extent of KRAS distribution to endomembranes, we calculated Manders’ coefficient, which measures the fraction of CellMask co-localized with GFP-KRASG12V (Cho et al., 2012b; Manders et al, 1993; Rehl et al, 2023). Our data show that while GFP-KRASG12V is predominantly localized to the PM in control cells, it is distributed intracellularly in the KD cell lines (Fig. 1B). Manders’ coefficients for CellMask are correspondingly higher in the KD cell lines, indicating that KRASG12V is redistributed to endomembranes after silencing *MTMR 2/3/4/7*. To directly quantify the extent of KRAS dissociation from the PM, intact basal PM sheets from T47D stably co-expressing GFP-KRASG12V and shRNA for *MTMR 2/3/4/7* were prepared and labeled with gold-conjugated anti-GFP antibodies and analyzed by electron microscopy (EM). We observed significant reductions in anti-GFP immunogold labeling after the KD, indicating loss of KRASG12V from the inner PM leaflet (Fig. 1C and S2). Spatial mapping of GFP-KRASG12V on the PM in *MTMR 2/3/4/7* KD cells revealed significant decreases in *L*_*max*_, the peak value of the *L(r)-r* clustering statistic that measures the extent of KRAS nanoclustering, which is essential for high-fidelity RAS signal transduction (Cho & Hancock, 2013; Cho et al, 2012a; Tian et al, 2007) (Fig. 1D). Confocal microscopy further demonstrates that HRASG12V cellular localization is not disrupted after silencing *MTMR 2/3/4/7* (Fig. S3). Together, our data show that MTMR 2/3/4/7 regulate the PM localization and nanoclustering of KRAS, but not HRAS.

### MTMR 2/3/4/7 regulate the cellular level and distribution of PtdSer.

**Figure 2.**
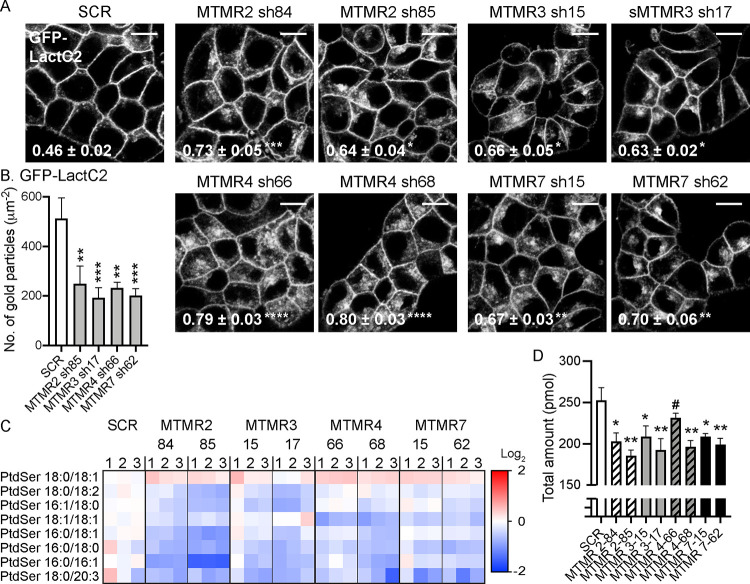
MTMR 2/3/4/7 regulate the cellular level and distribution of PtdSer. (**A**) T47D cells stably expressing GFP-LactC2 were infected with lentivirus expressing scrambled shRNA (SCR) or shRNA targeting *MTMR2, 3, 4* or *7*, followed by 1 ug/mL puromycin selection. Cells were incubated with CellMask for 1 h at 37°C incubator, fixed with 4% PFA and imaged by confocal microscopy. Representative GFP-LactC2 images are shown. Their corresponding CellMask and merged images are shown in Fig. S5. The inserted values represent a mean fraction ± S.E.M. of CellMask colocalizing with GFP-LactC2 calculated by Manders’ coefficient from three independent experiments. Scale bar – 10 μm. (**B**) Intact basal PM sheets prepared from T47D cells co-expressing GFP-LactC2 and shRNA targeting *MTMR2, 3, 4* or *7* were labeled with antiGFP-conjugated gold particles and visualized by EM. The graphs show a mean number of gold particles ± SEM (n ≥ 15). (**C**) Whole cell PtdSer levels were measured in these cells via electron spray ionization and MS/MS from three independent experiments. A heat map was constructed to quantify the changes of different lipid species after *MTMR* knockdown. Each row represents a different species of PtdSer, while each column represents a single sample. The scaled expression values of each lipid measured is plotted in red-blue color log_2_ scale. In relation to control (SCR) cells, red and blue colored tiles indicate higher and lower lipid contents, respectively. (**D**) The graph shows the mean of total moles ± S.E.M. of PtdSer. Significant differences between control (SCR-expressing) and *MTMR*-silenced cells were assessed using one-way ANOVA tests (* p < 0.05, ** p < 0.01, *** p < 0.001, **** p < 0.0001, # – not significant).

The PM interaction of KRAS, but not HRAS, is dependent on PM PtdSer content (Cho et al., 2012b; Cho et al, 2016b; Garrido et al., 2020; Miller et al., 2019; Tan et al., 2018; van der Hoeven et al., 2018; Zhou et al., 2017). To quantify PM PtdSer content, T47D cells stably co-expressing GFP-LactC2, a well-characterized PtdSer probe (Yeung et al, 2008), and shRNA targeting *MTMR 2/3/4/7* were incubated with CellMask and imaged by confocal microscopy. GFP-LactC2 is primarily localized to the PM in control cells but is redistributed in *MTMR 2/3/4/7* KD cells as reflected by higher Manders’ coefficient values (Fig. 2A and S4). EM analysis of isolated intact PM sheets prepared from T47D cells expressing GFP-LactC2 and incubated with anti-GFP antibody-conjugated gold shows that gold particle labeling density of the inner basal PM is significantly reduced when cells co-express shRNAs for *MTMR 2/3/4/7,* indicating depletion of inner PM PtdSer (Fig. 2B). The loss of inner leaflet PtdSer was not due to flipping to the outer leaflet since Annexin V binding to the outer PM leaflet, as measured by flow cytometry, did not increase in *MTMR 2/3/4/7* KD cells (Fig. S5). Finally, whole cell lipidomics revealed that *MTMR 2/3/4/7* KD resulted in a significant reduction in total cellular PtdSer content (Figs. 2C and D). Together, these results show that MTMR 2/3/4/7 potently regulates the PM and total levels of PtdSer; consistent with a mechanistic role for MTMR 2/3/4/7 in controlling PtdSer cellular distribution.

### MTMR 2/3/4/7 regulate PI4P content at the PM.

**Figure 3.**
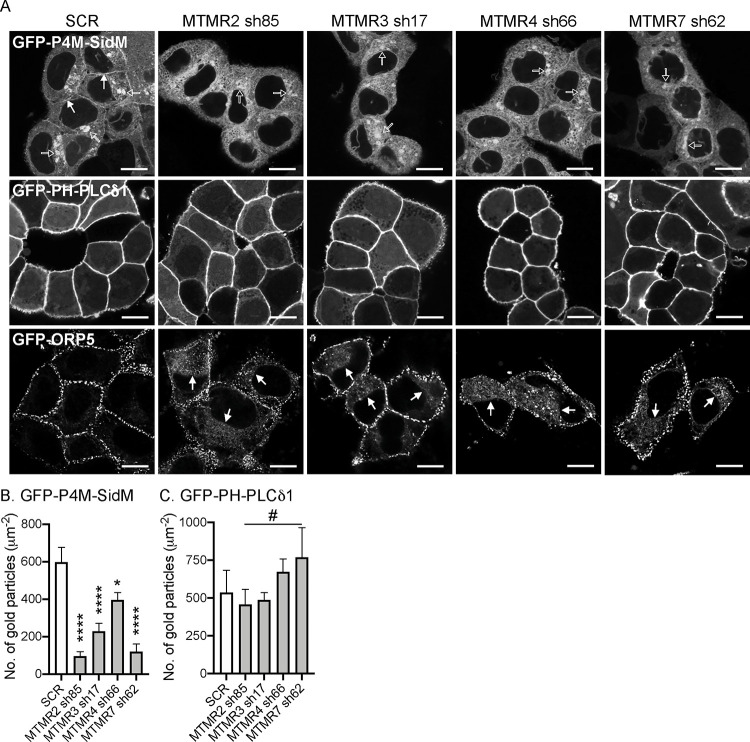
MTMR 2/3/4/7 regulate the localization of PM PI4P and ORP5. (**A**) WT T47D cells were infected with lentivirus expressing scrambled shRNA (SCR) or shRNA targeting *MTMR2, 3, 4* or *7*, followed by 1 ug/mL puromycin selection. These cells were transfected with GFP-P4M-SidM, -PH-PLCδ1, or -ORP5, and fixed with 4% PFA and imaged by confocal microscopy. Closed and open arrowheads for GFP-P4M-SidM indicate the staining of PM and Golgi complex, respectively. Closed arrowheads for GFP-ORP5 indicate the ER localization of ORP5. Scale bar – 10 μm. Intact basal PM sheet of Caco-2 cells co-expressing GFP-P4M-SidM (**B**) or –PH-PLCδ1 (**C**) with shRNA targeting *MTMR2, 3, 4,* or *7* were labeled with anti-GFP-conjugated gold particles and visualized by EM. The graphs show a mean number of gold particles ± SEM (n ≥ 15). Significant differences between control (SCR-expressing) and *MTMR*-silenced cells were assessed by one-way ANOVA tests (* p < 0.05, **** p < 0.0001, # – not significant).

In mammalian cells, inner PM leaflet PI4P and PI(4,5)P_2_ recruit the LTPs ORP5 and ORP8, to ER-PM membrane contact sites (MCSs) to exchange newly synthesized PtdSer in the ER for PI4P from the PM (Chung et al., 2015; Ghai et al, 2017; Moser von Filseck et al., 2015; Sohn et al., 2018). Depleting PM PI4P or PI(4,5)P_2_, concordantly depletes PtdSer and KRAS from the PM (Gulyas et al., 2017; Kattan et al., 2019). Depletion of PI4P at the Golgi complex also mislocalizes PtdSer and KRAS from the PM via an as yet unknown mechanism (Miller et al., 2019). To examine whether silencing *MTMR 2/3/4/7* perturbs cellular localization of PI4P and PI(4,5)P_2_, *MTMR 2/3/4/7* KD T47D cells expressing GFP-P4M-SidM or GFP-PH-PLCδ1, probes for PI4P (Hammond et al, 2014) and PI(4,5)P_2_ (Varnai & Balla, 1998), respectively, were imaged by confocal microscopy. GFP-P4M-SidM decorated the PM and Golgi complex in control cells (closed and open arrowheads, respectively in Fig. 3A), consistent with previous studies (Hammond et al., 2014; Miller et al., 2019), whereas *MTMR 2/3/4/7* KD perturbed GFP-P4M-SidM staining of the PM, but not the Golgi complex (Fig. 3A). By contrast, the PM localization of GFP-PH-PLCδ1 was unchanged in *MTMR 2/3/4/7* KD cells (Fig. 3A). EM analysis of immunogold labelled PM sheets also shows significant reduction in PI4P PM content, but no change in PI(4,5)P_2_ PM content after *MTMR 2/3/4/7* KD (Fig. 3B). Given the reduction in PM PI4P content, we next examined ORP5 cellular localization. Confocal imaging showed that GFP-ORP5 predominantly localized to the ER-PM MCS in control cells, but substantially redistributed, presumably, to the ER, in *MTMR 2/3/4/7* KD cells (Fig. 3A) (Du et al, 2020; Monteiro-Cardoso et al, 2022). We conclude that MTMR 2/3/4/7 regulate ORP5 localization to the ER-PM MCS by maintaining the PM level of PI4P.

### *MTMR 2/3/4/7* KD elevates PM PI3P content.

**Figure 4.**
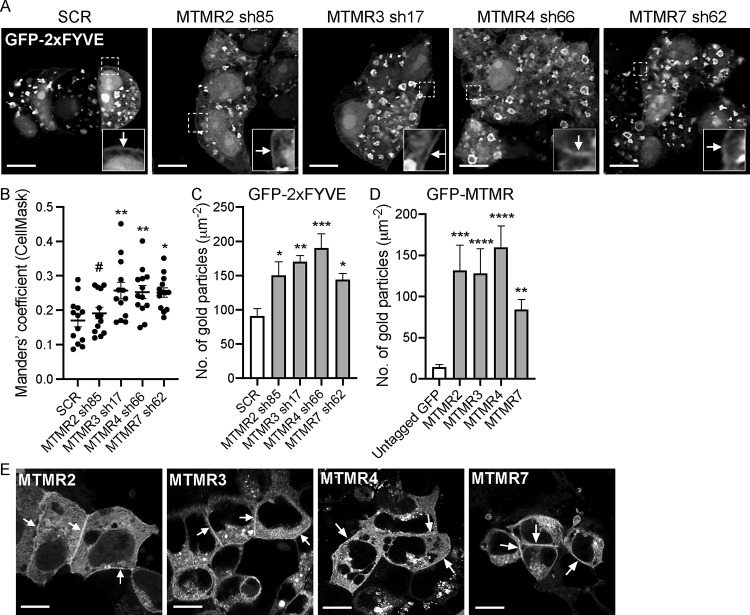
MTMR 2/3/4/7 regulate cellular PI3P contents. (**A**) T47D cells expressing scrambled shRNA (SCR) or shRNA targeting *MTMR2, 3, 4* or *7*, followed by 1 ug/mL puromycin selection, were overexpressed with GFP-2xFYVE. Cells were incubated with CellMask for 1 h at 37°C incubator, fixed with 4% PFA and imaged by confocal microscopy. Representative GFP-2xFYVE images are shown. Their corresponding CellMask and merged images are shown in Fig. S7. A selected region (the white square) is shown at a higher magnification. Arrowheads indicate the PM-staining of GFP-2xFYVE. (**B**) The graph represents a mean fraction ± S.E.M (n = 13) of CellMask colocalizing with GFP-2xFYVE calculated by Manders’ coefficient (**C**) Intact basal PM sheets prepared from these T47D cells from (A), or WT T47D cells expressing GFP-MTMR proteins (**D**) were labeled with anti-GFP-conjugated gold particles and visualized by EM. The graphs show a mean number of gold particles ± SEM (n ≥ 15). (**E**) Cells from (D) were fixed with 4% PFA and imaged by confocal microscopy. Scale bar – 10 μm. Arrowheads indicate the PM localization of MTMR proteins. Significant differences between control (SCR-expressing) and *MTMR*-silenced cells were assessed by one-way ANOVA test for (B and C) (* p < 0.05, ** p < 0.01, *** p < 0.001, **** p < 0.0001, # - not significant). Significant differences between untagged GFP (control) and GFP-MTMR-expressing cells were assessed by one-way ANOVA test for (D) (** p < 0.01, *** p < 0.001, **** p < 0.0001).

PI3P is synthesized from PI by class II and III PI3Ks, whence it can be converted to PI(3,5)P_2_ or PI by PI 5-kinase, or myotubularin (MTM) family phosphatases, respectively (Jean & Kiger, 2014; Pulido et al, 2013). To examine if depleting MTMR 2/3/4/7 alters cellular PI3P levels or distribution, *MTMR 2/3/4/7* KD T47D cells expressing the well-characterized PI3P probe, GFP-2xFYVE, the tandem FYVE domain of Hrs (hepatocyte growth factor-regulated tyrosine kinase substrate) (Gillooly et al, 2000) were stained with CellMask and imaged by confocal microscopy. Mander’s coefficient values estimating fractions of endomembranes co-localized with GFP-2xFYVE-decorated endosomes are elevated in *MTMR 3/4/7*, but not *MTMR2*, KD cells, suggesting increased PI3P content in early endosomes (Fig. 4A and B). Moreover, our data show some PM decoration with GFP-2xFYVE in all cells, indicative of PI3P at the PM (arrowheads in Fig. 4A). To formally quantify PM PI3P content, we performed EM analysis on intact basal PM sheets prepared from *MTMR 2/3/4/7* KD T47D cells expressing GFP-2xFYVE. The data reveal that silencing *MTMR 2/3/4/7* significantly elevates PI3P levels in the PM (Fig. 4C). Intriguingly, confocal and EM imaging further show GFP-MTMR 2/3/4/7 localization to the PM (Fig. 4D and E). Together, our data suggest that MTMR 2/3/4/7 localize to the PM, such that genetic depletion disrupts PI3P conversion to PI at the PM, which increases PI3P content at the PM and early endosomes.

### MTMR 2/3/4/7 regulate KRAS signaling.

**Figure 5.**
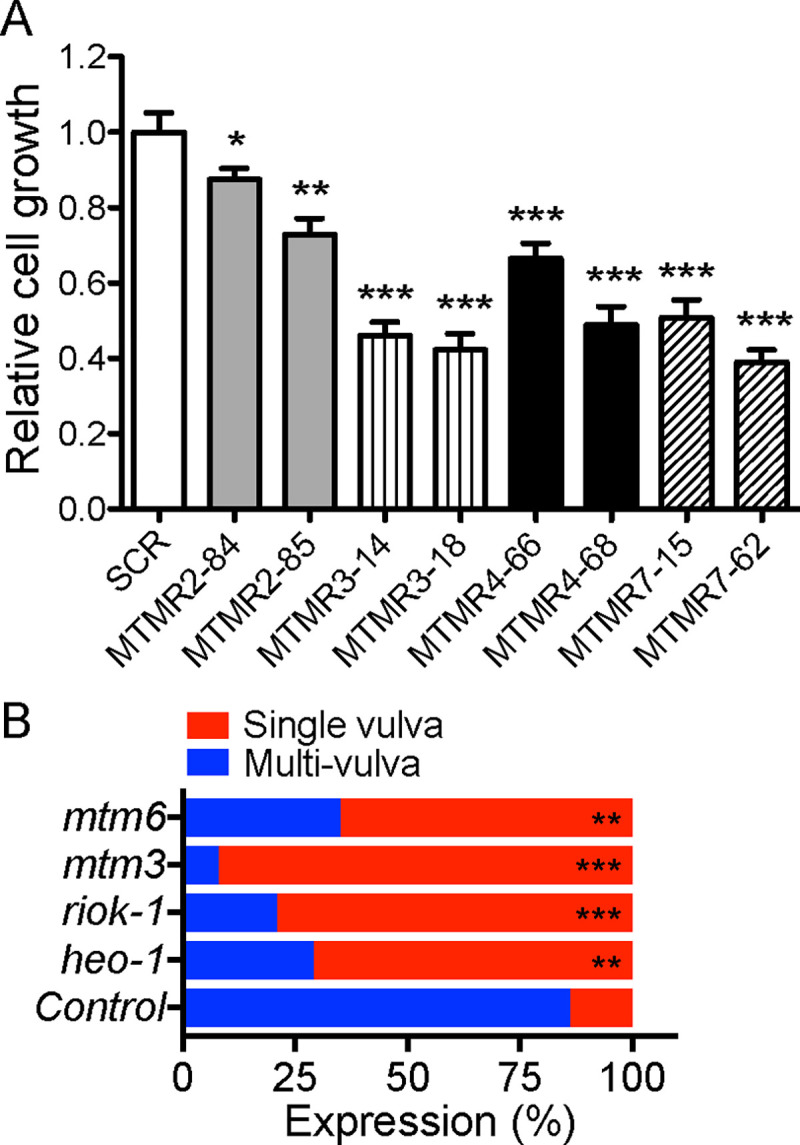
MTMR 2/3/4/7 regulate cell growth and *C. elegans* transformed by oncogenic KRAS. (**A**) T47D cells stably expressing GFP-KRASG12V were infected with lentivirus expressing scrambled shRNA (SCR) or shRNA targeting *MTMR2, 3, 4* or *7*, followed by 1 ug/mL puromycin selection. Cells were seeded on a 96-well plate and cultured for 4 days. Complete growth medium was replaced every 24 h. Cell numbers were counted to measure cell proliferation. The graph shows the mean cell proliferation ± S.E.M. from three independent experiments relative to that for the control cells (SCR-expressing). Significant differences between control and *MTMR* knockdown cells were assessed using one-way ANOVA test (* p<0.05, ** p<0.01, *** p<0.001). (**B**) RNAi was induced by feeding *let-60* (n1046) L1 worms through adult stage with *E. coli* strain HT115, producing dsRNA to target genes. The presence of the multi-vulva phenotype was scored using DIC/Nomarski microscopy. 100 – 200 worms were assayed per RNAi knockdown. Significant differences were evaluated in Student’s *t-*tests (** p < 0.01 and *** p < 0.001).

Since *MTMR 2/3/4/7* KD mislocalizes KRASG12V from the PM, we used two biological systems to evaluate effects on KRAS signal transmission. First, we examined KRAS-transformed T47D cells. Proliferation of *MTMR 2/3/4/7* KD cells stably expressing GFP-KRASG12V was significantly inhibited compared to parental cells replete for each *MTMR* gene (Fig. 5A). Secondly, we analyzed signaling by the KRAS ortholog, let-60 in the invertebrate model system *Caenorhabditis elegans* (*C. elegans*), where constitutively active mutation of *let-60* induces a quantifiable multivulva phenotype (Reiner et al, 2008). We used RNAi designed to target *C. elegans* MTMR orthologs and examined whether *MTMR* KD could suppress the multivulva phenotype associated with expression of LET-60 G13D (n1046). Our data show that silencing *mtm-3* (ortholog of *MTMR3*), or *mtm-6* (ortholog of *MTMR7*) abrogated multivulva formation, and indeed were equipotent with two previously reported potent suppressors of LET-60 G13D signaling (*heo-1* and *riok-1*) (Smith & Levitan, 2004; Weinberg et al, 2014) (Fig. 5B). Together, these data provide strong evidence that the MTMR phosphatases regulate KRAS signal transmission by maintaining PtdSer and KRAS PM localization.

## Discussion

We have identified new roles for the phosphatases, MTMR 2/3/4/7, in maintaining the PM localization of PI4P, PtdSer and KRAS. We show that KD of *MTMR 2/3/4/7* dissociates PtdSer and KRAS from the PM and also reduces total cellular PtdSer level. Concomitantly, *MTMR 2/3/4/7* KD elevates PM PI3P content, reduces PM PI4P content and dissociates ORP5 from the ER-PM MCS. Collecting these observations together, we propose that the PM-localized MTMR phosphatases are required to help maintain PM levels of PI for generating PM PI4P by PI4KA, which in turn maintains total PtdSer levels and drives ER to PM transport of PtdSer. PI is synthesized from phosphatidic acid at the cytosolic face of ER, where a fraction of PI is used for the synthesis of glycosylphosphatidylinositol-anchored proteins (Blunsom & Cockcroft, 2020), with the remaining PI being widely distributed across intracellular cytosolic membranes (Ashlin et al, 2021; Pemberton et al, 2020; Zewe et al, 2020). PI delivered from the ER directly to the PM by the LTPs, Nir2/3 and TMEM24 (Posor et al, 2022), is a crucial substrate for PM polyphosphoinositides (PPIn). Simple overexpression of Nir2/3 and TMEM24 elevates the synthesis and total amount of PM PPIn (Chang et al, 2013; Kim et al, 2015; Lees et al, 2017), suggesting that PM PI is constantly converted to PPIn and the PI supply to the PM is rate-limiting. In this context, the pathway to PI generation from PI3P by MTMR 2/3/4/7 at the PM, emerges as a critical alternative supplier of PI to allow sufficient PM PI4P generation hence PtdSer transport by the ORP5/8 machinery (Fig. 5C). In addition to the PM, MTMR 2/3/4/7 localize to endosomes and promotes their trafficking by regulating endosomal PI3P and PI(3,5)P_2_ levels (Bonneick et al, 2005; Franklin et al., 2011; Mochizuki & Majerus, 2003; Naughtin et al., 2010; Previtali et al, 2007; Walker et al., 2001). Thus, perturbing endosomal trafficking by *MTMR 2/3/4/7* KD will block the delivery of endosomal PI to the PM, further contributing to the reduced PM PI content. Therefore, when *MTMR 2/3/4/7* is depleted, PM PtdSer level falls and KRAS dissociates from the PM (Fig. 5C). We did not investigate the mechanism whereby *MTMR 2/3/4/7* KD reduced total cellular PtdSer content, but previous work has shown that PM PI4P depletion indirectly blocks PtdSer synthase 1 and 2 activities (Sohn et al, 2016). Since PM PI4P levels were reduced in *MTMR 2/3/4/7* KD cells, the same mechanism is likely responsible.

Inactivating mutations in *MTMR2* are found in Charcot-Marie-Tooth disease type 4B1, a severe autosomal recessive neuropathy with demyelination and myelin outfoldings of the nerve. Studies have proposed that disrupted endosomal trafficking by elevating endosomal PI3P and PI(3,5)P_2_ due to inactive MTMR2 likely contributes to the demyelination of nerve cells (Bonneick et al., 2005; Previtali et al., 2007). Intriguingly, PtdSer is the main component of myelin sheath (Ma et al, 2022), and inhibition of PI4KA in Schwann cells reduces PM and total levels of PtdSer and PI4P, and induces aberrant myelination (Alvarez-Prats et al, 2018). Thus, in addition to disrupted endosomal trafficking by *MTMR2* inactivation, it is plausible that the reduced PM levels of PtdSer and PI4P further contributes to the demyelination observed in Charcot-Marie-Tooth disease type 4B1.

In sum, our study proposes a new role for the MTMR phosphatases in maintaining PM PI4P and PtdSer contents by maintaining an adequate supply of PM PI from PI3P. Depleting MTMR 2/3/4/7 in turn, reduces PM and total cellular PtdSer levels, blocking KRAS PM localization and KRAS signaling. Thus, the mechanisms that maintain PM PI content may be contain useful targets to abrogate oncogenic KRAS signaling.

## Figures and Tables

**Figure 6.**
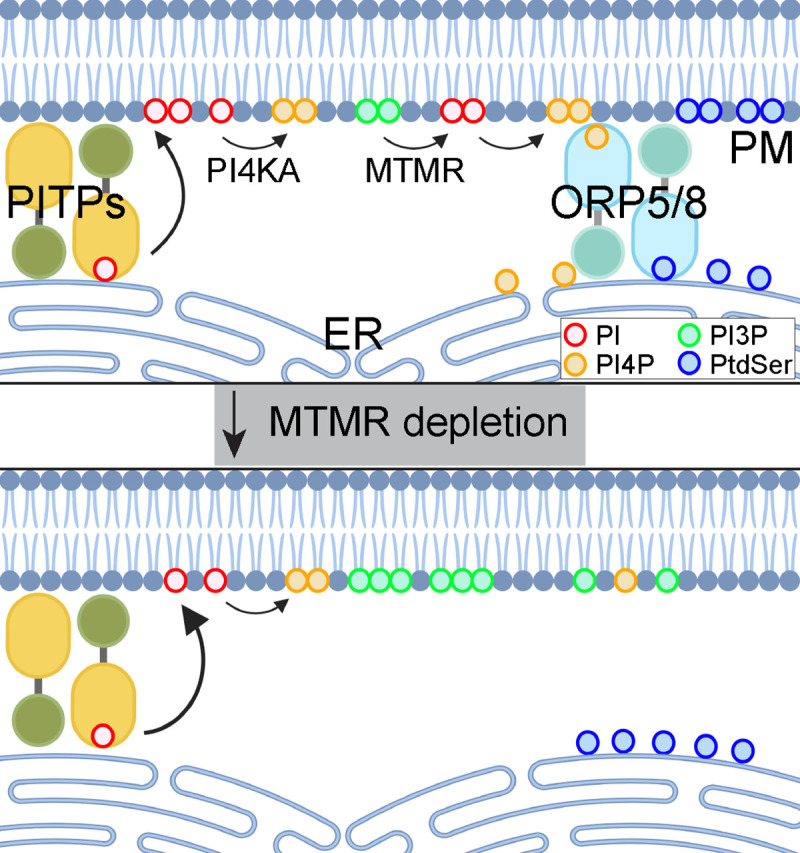
A working model of our study. PITPs deliver newly synthesized PI from the ER to PM, and MTMR at the PM concomitantly converts PI3P to PI, which are which are crucial substrate for PM PPIn like PI4P. ORP5 and 8, the lipid transfer proteins for PtdSer, exchanges ER PtdSer with PM PI4P at ER-PM membrane-contacting sites, which enriches PM PtdSer contents. This, in turn, allows stable PM binding of KRAS, and thereby KRAS signal output. When MTMR is depleted, PM PI3P conversion to PI is blocked, providing insufficient PM PI contents for PI4P synthesis. This dissociates ORP5/8 from ER-PM membrane-contacting sites, resulting in reduced PM localization of PtdSer and thereby KRAS. PITPs – PI transfer proteins, PI4KA – phosphatidylinositol 4-kinase IIIα, ORP – oxysterol-binding protein related protein, PM – plasma membrane, ER – endoplasmic reticulum.
